# Consumer awareness and production practices of farmers on antimicrobial residues in chicken eggs and Chinese cabbage in Dodoma, Central Tanzania

**DOI:** 10.1371/journal.pone.0272763

**Published:** 2022-08-18

**Authors:** Richard John Mongi, Eugene Benjamin Meshi, Julius Edward Ntwenya

**Affiliations:** Department of Public Health and Community Nursing, University of Dodoma, Dodoma, Tanzania; West Bengal University of Animal and Fishery Sciences, INDIA

## Abstract

**Background:**

Antimicrobial residues (ABs) in foods contribute to the development of antimicrobial resistance, which is becoming a major public health concern around the world. Understanding food production practices concerning antimicrobial use and consumer awareness on the possibility of ABs in foods is necessary for developing mitigation strategies. Therefore, this study was conducted to assess the production practices and awareness among eggs and Chinese cabbage consumers in Dodoma city.

**Methods:**

A cross-sectional study was conducted using a structured questionnaire and checklist to collect data on awareness and production practices from 420 consumers, 30 chicken egg farmers, and 30 Chinese cabbage farmers in eight city wards.

**Findings:**

About 42% of consumers of eggs and Chinese cabbages were not aware of the likelihood of antimicrobial residues in these foods. The awareness was significantly influenced by the consumer’s educational level (p = 0.001) and geographical location (p = 0.045), with educated and urban consumers being 7.7 and 1.6 times more informed than their uneducated and peri-urban counterparts. Furthermore, the majority of chicken farmers raised more layers than broilers, and they all used antimicrobial drugs, mainly tetracycline and its variants, to treat and prevent diseases. The findings also revealed that many vegetable producers utilized wastes from animals treated with antimicrobials as manure to amend the soil.

**Conclusion:**

In a view of the findings, a considerable number of egg and Chinese cabbage consumers in Dodoma city were unaware on the likelihood of antimicrobial residues in these foods from their production practices. All chicken farmers utilized antimicrobial drugs mainly tetracycline and its derivatives, for the treatment and prevention of diseases while vegetable producers used wastes from animals treated with antimicrobials as soil manure. Therefore, initiatives to inform farmers and consumers about the possibility of antimicrobial residues in these foods and their related public health risks upon long-term consumption are strongly advised.

## Introduction

Antimicrobial residues in food have gained a lot of attention in recent years as food safety and public health concerns have grown. These residues can be found in milk, eggs, and meat due to the indiscriminate use of antimicrobials in animal production for various reasons, including therapy, disease prevention, and growth promotion [1]. Moreover, plant-derived foods grown in soil and water contaminated with animal waste containing antimicrobial residues may secondarily expose humans to veterinary antimicrobials with consequent antimicrobial resistanc60 farmers (30e [2]. A large fraction of antimicrobial drugs are not metabolized in the animal body and are expelled as waste into the soil in their original states or as active metabolites via urine or feces [3]. The wastes are often employed as manures for agricultural production, which enhances their likelihood of being uptaken by plants [4]. It has been reported that antimicrobial residues in edible animal products have expanded above permitted limits in developing countries including Tanzania, posing a risk to human health [5, 6]. According to a study by Nonga et al. [7] in Morogoro, Tanzania, antibiotics account for 85% of the drugs used in the farms by volume, and their usage is marked by overprescribing, abuse, and/or misuse in intensive layer farms, resulting in pronounced residues in chicken eggs. Tetracyclines are among the most commonly utilized antimicrobials [4, 7, 8], followed by beta-lactam antibiotics such as penicillins [9, 10].

Antimicrobial residues are linked to the development and spread of antimicrobial resistance, which is a major public health concern around the world [5, 11, 12]. Many aspects of life are jeopardized by antimicrobial resistance, including health care, food security, sanitation and safe drinking water, as well as income gaps [13]. According to O’Neil [14] and world bank [15] reports, yearly global fatalities from antibiotic-resistant illnesses are expected to rise from 700,000 in 2014 to 10 million by 2050, resulting in billions of dollars in healthcare expenditures and an economic cost of trillions of dollars. Antimicrobial resistance is also expected to push up to 24 million people into extreme poverty globally by 2030 [16]. Human problems linked to AMR include higher healthcare expenses as well as higher morbidity and mortality rates [5, 11, 12], calling for collaboration among all sectors and stakeholders, including farmers and consumers, to combat the problem. Despite the Tanzania government’s efforts through the 2017–2022 National Action Plan [17] and other researchers [7–9] efforts to address the growing threat of antimicrobial residues and their associated resistance in Tanzania, farmers and food consumers are still not explicitly involved in tackling the problem.

Chicken eggs and Chinese cabbage are among the most common meals in Tanzania, where yearly production is 4.05 billion eggs and per capita consumption was 106 eggs per year in 2020 which is expected to rise to 202 eggs per year in 2050 [18]. An average of 3.4 tons per hectare of Chinese cabbage has been reported to be produced in Dodoma city each year [19]. As previously reported [4, 5, 6], production practices for these foods involve direct and indirect use of antimicrobial agents, which may result in harmful residues. Unfortunately, consumers in Tanzania, like those in many other poor countries, are unaware of their basic rights as well as the quality and safety of the food they buy and consume [20]. As a result, assessing and raising consumer awareness is crucial to resolving the situation, as it will allow them to make informed purchases and force processors and sellers to follow regulations. Despite a literature search, there is minimal information on consumer knowledge of antimicrobial residues and production techniques among chicken egg and Chinese cabbage farmers in Dodoma, Tanzania. The objective of this study was to investigate and provide this missing data that may be utilized to plan and mitigate the problem of foodborne antimicrobial residues and thereby protect consumers’ health in Dodoma city and across the country at large.

## Materials and methods

### Study area

The study was conducted in Dodoma City which is the national capital of Tanzania and its selection has been based on its fast population growth attributed to the government’s decision to move its business to the City. More importantly, no study has ever been conducted to assess the missing information on this growing population.

### Study design

Cross sectional survey was considered to assess production practices used by farmers in raising chickens and cultivating vegetables as well as the level of awareness of foodborne antimicrobial residues among consumers in these foods across two weeks in March 2021. Consumers who were 18 years and above were eligible and included in the study.

### Sample size and sampling procedure

#### Subjects for consumers’ awareness

A total of 422 subjects were considered for the study from a total population of 410, 956 in the city using a formula by Fisher [21] as depicted in Eq 1.


$$\begin{array}{l} n=N*X/(X+N–1),\;where,\\ X=Z_{\alpha /2}{}^{2}*p*(1‐p)/MOE^{2}, \end{array}$$ (1)

Z_α/2_ is the critical value of the Normal distribution at α/2 = 1.96), MOE is the margin of error = 5%, p is the sample proportion of 50%, the p is not known therefore is estimated to be 50% and N is the population size 410, 956. The sample size was 384 subjects and the attrition rate was estimated to be 10%, therefore the final sample size estimate was (0.1 x 384) + 384 = 422. However, only 420 subjects participated in the study.

### Sampling technique

**Fig 1:**
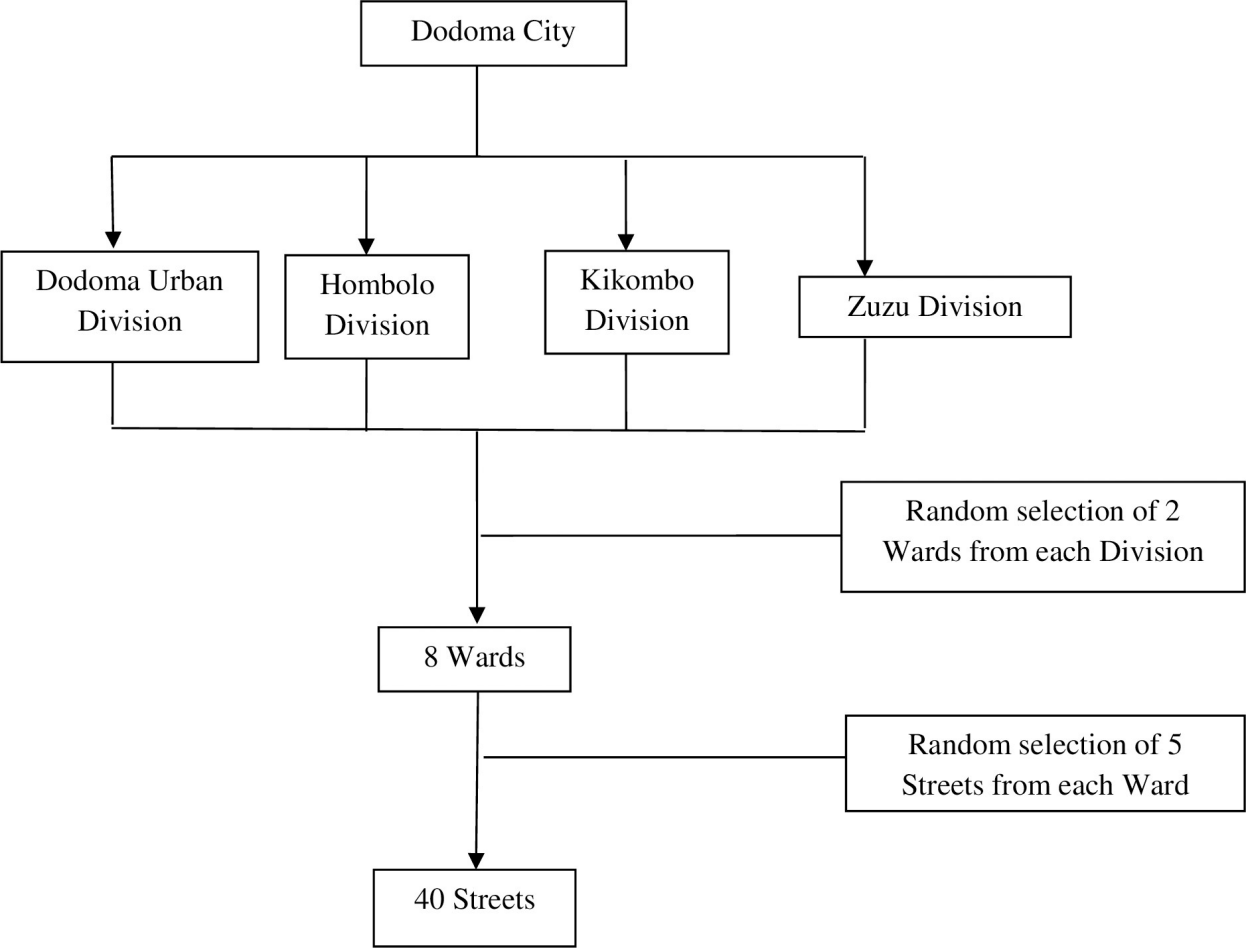
Selection of wards and streets for the study.

A multistage random sampling technique was used to obtain representative wards and streets as described by Kothari [22]. All four city divisions were conveniently included in the study, and the sample was equally distributed to all streets. From each division, two wards were randomly selected from which five streets were selected, making a total of 40 streets as depicted in Fig 1. A simple random selection of subjects was conducted at each street to get a representative sample for the study. The selected wards were Kisasa, Nkuhungu, Kikuyu North, Nzuguni, Mtumba, Ngh’ongh’onha, Hombolo-bwawani, Mpunguzi, Zuzu, Miyuji, and Makutopora.

#### Selection of eggs and vegetable farmers

About 60 farmers (30 for eggs and 30 for Chinese cabbage) were randomly selected from the list provided by the city extension office. The selection of egg farmers was based on their consent to participate in the study as well as the presence of chickens, while the selection of vegetable farmers was based on consent, the presence of vegetable gardens, and the usage of animal manure from commercial animal farms during that period.

### Data collection

#### Consumers’ awareness of antimicrobial residues

A structured questionnaire with closed and open-ended questions was used to collect data on consumer awareness through face-to-face interviews. The questionnaires were pre-tested for reliability and validity on 5% of the sample size after being translated from English to Swahili (the national language). The pretest was conducted in two wards that were not included in the study (Ihumwa and Dodoma Makulu wards). The questionnaire was then revised for wording, sequencing, and contents as per the results of the pretest. The English version of the questionnaire has been provided as a supporting document (S2 File). Data were collected by trained data collectors under close supervision of the researchers.

#### Production practices of eggs and Chinese cabbage

Farmers’ production methods were collected via face-to-face interviews using a checklist developed by Kline et al. [23]. Questions on field management, manure application and management, record keeping, and irrigation water were included in the checklist, which was pre-tested among farmers before being assessed for reliability and validity.

### Data analysis

The data were analyzed using SPSS Statistics 25.0 for Windows (SPSS Inc., Chicago, IL, USA). Descriptive statistical studies, such as frequency and percentage and inferential statists were both conducted. Each participant’s awareness responses were converted to numerical scores and a score out of seven (7) was assigned based on the number of "yes" responses across the seven (7) categories [24]. The Cronbach’s alpha value of the seven items used to assess awareness of antimicrobial residue was 0.70, which is within an acceptable range. Binary logistic regression analysis was performed to assess the influence of social-demographic characteristics on the participants’ level of awareness at p < 0.05 and the 95% confidence interval (CI) for an adjusted odds ratio (AOR).

### Ethical considerations

The proposal was reviewed and approved by the University of Dodoma’s Ethical Review Committee (Reference number MA.84/261/02). The Dodoma City Director and Regional Commissioner issued the data collection permits. Written consent was sought from all study participants over the age of 18 years after explaining the study objectives, procedures, and the confidentiality of the data. They were guaranteed the opportunity to withdraw from participating in the study at any moment.

## Results

### Consumer awareness on the likelihood of antimicrobial residues in eggs and Chinese cabbage

#### Demographic information of respondents

The majority (64.5%) of the total 420 participants were from urban wards and more than (54.3%) were men with the majority of them being less than 35 years old. A high proportion (70.2%) of participants had completed primary and secondary school and about 44% of them engage themselves in the business sector (Table 1).

**Table 1:** Demographic information of respondents.

Variable	Frequency (n)	Proportion (%)	95% CI
**Location**			
Urban	270	64.3	59.5–68.9
Peri-urban	150	35.7	31.1–40.5
**Gender**			
Male	228	54.3	49.4–59.1
Female	192	45.7	40.9–50.6
**Age group**			
<35 years	257	61.2	52.4–65.9
> 35 years	163	38.3	34.1–43.7
**Education**			
No education	33	7.9	5.5–10.9
Primary	139	33.1	28.6–37.8
Secondary	156	37.1	32.5–41.9
Tertiary	66	15.7	12.4–19.5
Vocational	26	6.2	4.1–8.9
**Occupation**			
Farmers	136	12.4	25.7–34.6
Business	185	44	39.2–48.9
Others	99	23.6	19.6–27.9

#### Consumption practices

The results of egg and Chinese cabbage consumption practices are depicted in Table 2. Markets were the main purchasing sources of both eggs and Chinese cabbage for consumption. Furthermore, most of them were reported to consume fried eggs and Chinese cabbage at least once per week at home in fried form.

**Table 2:** Eggs and Chinese cabbage consumption practices.

Variable	Eggs (n = 420)		Chinese cabbage (n = 420)	
	Frequency (n)	Percent (%)	Frequency (n)	Percent (%)
**Source**				
**Open market**	128	30.5	200	47.6
**Supermarket**	18	4.3	6	1.4
**Farm**	57	13.6	69	16.4
**Shops**	117	27.9	45	10.7
**Street markets (*Gengeni*)**	100	23.8	100	23.8
**Consumption frequency**				
**Once/day**	70	16.7	146	34.8
**Once /week**	180	42.9	206	49.0
**Once /month**	105	25.0	36	8.6
**Once/year**	24	5.7	5	1.2
**Don’t consume**	41	9.7	27	6.4
**Consumption form**				
**Raw**	19	4.5	3	.7
**Fried**	228	54.3	267	63.6
**Boiled**	173	41.2	150	35.7
**Consumption place**				
**At home**	351	83.6	380	90.5
**Hotel/restaurant**	64	15.2	40	9.5
**Street**	5	1.2	0	0

#### Consumers’ awareness of the likelihood of antimicrobial residues in eggs and Chinese cabbage

Table 3 depicts consumer awareness of antimicrobial use in animal production, as well as residues in eggs and Chinese cabbage. Overall, 58% of consumers were aware of the possibility of antimicrobial residues in eggs and Chinese cabbages while 42% were not. Specifically, the majority of them were aware of the use of antimicrobial drugs to treat sick animals (80.7%) and the possibility of drug residues causing harmful effects on humans (77.6%). On the other hand, only a few consumers were aware of the possibility of drug residues in vegetables, common antibacterial agents that could result in drug residues in foods, and preventative strategies. Furthermore, despite the fact that the majority (17.6%) of participants were aware of prevention strategies, an appreciable number (41.9%) mentioned cleaning as one of them, showing a lack of understanding in this area.

**Table 3:** Consumer’s awareness of drug usage in animal production and preventive methods of antimicrobial residues in eggs and Chinese cabbage.

Awareness item	Response (n = 420)		
	Frequency (n)	Proportion (%)	95% CI
Overall awareness on the likelihood of antimicrobial residues in eggs and Chinese cabbage	243	58	53.0–62.6
Animals are treated with antimicrobial drugs when are sick	339	80.7	76.6–84.4
Heard about drugs residue in the food of animals and plants’ origin	236	56.4	51.3–61.0
Possibility of drug residues in vegetables	114	27.1	23.0–31.7
Possibility of drug residues to cause harmful effects on human	326	77.6	73.3–81.5
Any common antimicrobial agent which can cause drugs residue in food of plants and animals origins	223	53.1	48.2–58.0
Animal waste/manure containing antimicrobial drugs is used for cultivating fruits and vegetables	306	72.9	68.3–77.1
Prevention of drug residues in eggs and vegetable	74	17.6	14.1–21.6
**Prevention methods (n = 74)**			
Cleaning	31	41.9	30.5–53.9
Cooking	5	6.7	2.2–15.1
Cooking and cleaning	11	14.8	7.7–25.1
Follow drugs instruction	10	13.5	6.7–23.5
Stop using antibiotics	8	10.8	4.8–20.2
Education to farmers	9	12.2	5.7–21.8

#### Relationship between social demographic information and awareness of the likelihood of antimicrobial residues in foods

The impact of socioeconomic demographic variables on consumer awareness of antimicrobial residues in eggs and Chinese cabbage is shown in Table 4. The respondents’ understanding of antimicrobial residues was significantly influenced by their educational background (p = 0.001) and geographic location (p = 0.045). Graduates were 7.7 times more aware of antimicrobial residues than those who had not attended school, whereas those who lived in urban wards were 1.6 times more conscious of antimicrobial residues than those who lived in peri-urban wards.

**Table 4:** Binary logistic regression analysis showing social demographic characteristics that affect consumer’s awareness.

Variable	Category	AOR [95% CI]	P - Value
**Age**		1.025 [0.988–1.064]	0.185
**Age Group**	<35 (Ref)	1.00	
	>35	.799 [.398–1.603]	0.528
**Sex**	Female (Ref)	1.00	
	Male	1.197 [0.793–1.809]	0.392
**Education**	Informal (Ref)	1.00	
	Primary	3.050 [1.305–7.127]	0.010**
	Secondary	3.753 [1.554–9.065]	0.003**
	Vocational	6.593 [2.351–18.484]	0.002**
	University	7.740 [2.246–26.679]	0.001**
**Location**	Rural (Ref)	1.00	
	Urban	1.552 [1.009–2.385]	0.045*
**Occupation**	Farmers (Ref)	1.00	
	Business	1.367 [.816–2.291]	0.235
	Others	1.649 [0.831–3.274]	0.153

AOR, adjusted odds ratio; CI, confidence interval. The asterisks (*) and (**) indicate a significant effect at p 0.05 and p 0.01 respectively.

### Respondents’ practices on antimicrobial residues

#### Characteristics of chicken production

Table 5 shows the farming practices of chickens in the study area. The majority (46.7%) of farmers kept layers for egg production, with most of them owning less than 1, 000 chickens under an intensive production system. Furthermore, the majority of farmers used animal waste as manure on their crops as their primary method of disposal.

**Table 5:** Farming characteristics of chicken production (n = 30).

Variable	Frequency (n)	Percent (%)	95% CI
**Breed**			
**Broiler**	6	20.0	7.7–38.6
**Layers**	14	46.7	28.3–65.7
**Both**	10	33.3	17.3–52.8
**Number of Chicken**			
**100–500**	10	33.3	17.3–52.8
**501–1000**	10	33.3	17.3–52.8
**> 1000**	10	33.3	17.3–52.8
**Production systems**			
**Intensive**	24	80	61.4–92.3
**Semi intensive**	6	20	7.7–38.6
**Waste disposal**			
**Use as manure**	28	93.3	77.9–99.2
**Around the farm**	2	6.7	0.8–22.1

#### Antimicrobial usage in poultry production

All chicken producers employed antimicrobial treatments, with the majority (80%) utilizing more than one medication (Table 6). Disease prevention and treatment were the main reasons for using antimicrobial medications in the production of poultry and their products, with veterinary doctors being the most credible sources of information. Tetracyclines were the most commonly utilized antimicrobial agents by all farmers, with oxytetracycline having the highest proportion (100%) followed by doxycycline and tetracycline. Other medicines were used less often, with only 27% of farmers using them.

**Table 6:** Antimicrobial usage in poultry production (n = 30).

Item	Response		
	Frequency (n)	Proportion (%)	95% CI
**Use of Antimicrobial agent**	30	100	88.4–100
**Drug combinations**	24	80	61.4–92.3
**Reason for antimicrobial usage**			
Disease prevention and treatment	14	46.7	28.3–65.7
Disease prevention	12	40.0	22.7–59.4
Prevention, treatment and growth promotion	4	13.3	3.8–30.7
**Type of antimicrobial agent used**			
Oxytetracycline (OTC)	30	100	88.4–100
Tetracycline	14	46.7	28.3–65.7
Doxycycline	16	53.3	34.3–71.7
Cotrimoxazole	8	26.7	12.3–45.9
Amoxyline	8	26.7	12.3–45.9
Metronidazole	6	20	7.7–38.6
Chloramphenicol	4	13.3	3.8–30.7
**Sources of Information on Antimicrobial agent**			
Veterinary doctor	18	60.0	40.6–77.3
By self	4	13.3	3.8–30.7
Seller	6	20.0	7.7–38.6
Friend	2	6.7	0.8–22.1
**Frequency of antibiotic use**			
Once in three months	6	20	7.7–38.6
When they are sick	24	80	61.4–92.3
**Method or antibiotic treatment**			
Water	22	73.3	54.1–87.7
Water and injection	4	13.3	3.8–30.7
Water and food	4	13.3	3.8–30.7
**Compliance with a withdrawal period**	18	60	40.6–77.3

#### Production practices of vegetable

Table 7 depicts the use of soil and manure in Chinese cabbage farming. The majority (60%) of farmers used raw manure to amend vegetable-growing soil, and many of the producing areas (66%) were close to or near livestock production areas. Furthermore, most farmers were not keeping the waste lagoon from leaking or the manure storage area contained to avoid contamination. Only less than half (46.7%) took steps to prevent animals from entering the crop-growing areas.

**Table 7:** Manure and water usage in Chinese cabbage production (n = 30).

Item	Response		
	Frequency (n)	Proportion (%)	95% CI
**Manure usage**			
What is used as a soil amendment			
Raw manure	18	60.0	40.6–77.3
Raw manure or a combination of raw and composted manure	6	20.0	7.7–38.6
Industrial fertilizers	6	20.0	7.7–38.6
Crop production near or adjacent to the animal production areas	20	66.7	47.2–82.7
Maintenance of manure lagoons to prevent leaking or overflowing	12	40.0	22.7–59.4
Containment of manure stored near crop production to prevent contamination	14	46.7	28.3–65.7
Restriction of wild and domestic animals from entering the crop production area	14	46.7	28.3–65.7
Testing of crop production areas that have been subjected to flooding for potential antimicrobial hazards.	0	0	
**Water usage**			
Pond	12	40.0	22.7–59.4
Well	14	46.7	28.3–65.7
Municipal	4	13.3	3.8–30.7
Restriction of livestock from accessing the source of crop irrigation	7	46.7	9.9–42.3

## Discussion

### Consumer awareness on the possibility of antimicrobial residues in eggs and Chinese cabbage

#### Consumption practices

Consumption of eggs and Chinese cabbage is common in Dodoma city as well as in other parts of the country [17, 18]. This suggests that if the produced eggs and vegetables contain antimicrobial residues originating from their production practices, then the observed high intake frequency may be sufficient to have a long term health impact.

#### Consumer awareness of the possibility of antimicrobial residues in eggs and Chinese cabbage

Consumer awareness is concerned with individual customers’ rights and obligations within the marketplace exchange process [25, 26]. The poor consumer awareness found in this study could be linked to a lack of knowledge, as the majority of the participants had only attended basic and secondary schools. This compromises their ability to make informed food choices against potentially unsafe foods, such as those containing antimicrobial residues, necessitating strategies to create awareness about the existence and health impact of antimicrobial residues in foods. Other studies [9, 27, 28] have found similarly low levels of public awareness regarding antimicrobial residues in foods. In addition, a lack of proper communication about antimicrobial residues and resistance to food consumers from informed people or institutions may have contributed to low awareness [28]. It is well-thought-out that including well-informed customers in the fight against antimicrobial residues in foods may accelerate its success since they will make informed purchases and force processors to adhere to antimicrobial usage rules during food production. In another study, Nabwire [29] found that education has a comparable large impact on honey consumer awareness. However, despite the positive influence of knowledge on consumer awareness However, despite the positive effect of information on consumer awareness of the potential for antimicrobial residues in the studies, food selection and consumption depend on a variety of other factors, including nutritional knowledge, availability, hunger, taste, appetite, preferences, culture, and economic variables like price and income as well as policy [30, 31]. For example, In low-income families where educated individuals may reside, the availability of food at a reasonable price is often the most important consideration when choosing food items, regardless of quality or safety content [32]. Additionally, sometimes there is a negative correlation between awareness or knowledge and actions. For instance, someone may be aware of antibiotic residues in food yet choose not to take action knowingly or unknowingly. This suggests, that attempts to reduce or eradicate antimicrobial residues in foods and their related health impacts should engage all members of society, regardless of their social status. Moreover, the observed effect of location on consumer awareness could be attributed to the differences in cultural, social, and demographic elements that characterize different setups. Pambo [33] found a similar higher degree of awareness among urban sugar consumers, whereas Ishak and Zabil [26] found a significantly lower level of consumer awareness among urban residents compared to rural residents.

### Eggs and Chinese cabbage production practices

Egg-laying chickens are the most common commercial breed in Dodoma, and they are mostly maintained by small-to medium-scale farmers with flocks of 200 to 2000 birds [34]. Farmers’ preference for layers over broilers may be attributed to the durability of their egg production, which is linked to a steady stream of income for the farmers, as previously reported [35, 36]. Furthermore, compared to broilers, layers require a low-cost feed with inferior nutritional quality for egg production [37]. In many urban and peri-urban settings, intensive and semi-intensive production systems are the most frequent commercial chicken production systems [38]. The efficiency, ease, and economies of the intensive production system could be linked to its high frequency in modern chicken production with large numbers [34]. On the other hand, intensive poultry raising systems necessitate consistent access to health care, which may include the use of substantial doses of antimicrobials for therapeutic, prophylactic, and growth promotion purposes [39]. A considerable amount of these agents are excreted into the environment via urine and used for soil amendments, and their presence around chicken enterprises has been recorded. According to Campagnolo et al. [40], antimicrobial compounds were found in 67% of groundwater and surface water resources near large poultry businesses in Ohio. Similar usage of animal dung as a soil amendment has been documented in other parts of the country [41].

A number of antimicrobial chemicals are used in commercial chicken raising in Dodoma city for therapeutic, preventive, and growth promotion purposes [39]. Despite antimicrobials’ preventive and curative functions, which help to improve the supply of animal products in the food chain [42] their widespread use and residues in foods like meat and eggs endanger human health [43]. The antimicrobials use in poultry farming with tetracycline species being the most common drugs agree with other studies [9, 27, 40]. Despite the fact that the majority of farmers (60%) claimed to get antimicrobial information from reliable sources like doctors, the proportion of misinformed farmers remains high, implying that these agents could be used indiscriminately in poultry farming. This violates the Veterinary Act No. 16 of 2003 [44], which regulates veterinarians and paraprofessionals, as well as the circumstances in which unqualified employees may perform some responsibilities. Engagement of non-professionals as sources of drug information and in treating animals, as well as many farmers’ failures to observe the mandatory withdrawal period, as seen in this study, could be linked to a lack of coordinated animal monitoring systems, lax or lenient implementation of antimicrobial use and food safety rules, a lack of basic awareness of antimicrobial usage in animal production and resistance among livestock keepers in Tanzania [10, 45–47]. As a result, antimicrobial overuse and abuse in food and agriculture production play a key part in the development and spread of AMR, as well as the public health implications that come with it [27].

The Tanzania Medicines and Medical Devices Authority (TMDA) is responsible for regulating Tanzania’s pharmaceutical sector, which is governed by the Food, Drugs, and Cosmetics Act of 2003 and the Tanzania Medicines and Medical Devices Act of 2019 [48]. The body regulates the quality, safety and effectiveness of medicines, medical devices, and diagnostics [49]. Moreover, the Veterinary Council of Tanzania (VCT) regulates and monitors the delivery of animal health services and the dispensing of veterinary medicines to consumers under the Veterinary Act No. 16 of 2003 [44]. However, the supply chain is driven by the private sector, and thus, it is common to find importers, distributors, and wholesalers delivering drugs directly to consumers via unofficial channels [50–52]. Generally, legislations regarding antibiotic drug application in farm animals as well as monitoring and control of their residues are not adequately enforced in the country [7]. Consequently, an overwhelming number of medicines sold in pharmacies in Tanzania are dispensed without a prescription, increasing the risk of unregulated antimicrobial sales for human and veterinary use [48, 53, 54]. This indicates that the country’s food safety regulatory institutions and related stakeholders should work together under one health approach to identify and implement appropriate mitigation solutions.

The use of waste from an animal treated with antimicrobials as manure for soil amendments in vegetable production is common in Dodoma city and other parts of the country [41]. However, a large proportion of antimicrobial agents are not metabolized in the body and are expelled as waste in the soil via urine or feces in an active form [3]. The interactions of these agents with bacteria in the environment may contribute to the formation of antimicrobial-resistant bacterial strains [2]. As a result, the widespread use of these wastes in agricultural activities increases the risk of antimicrobial residues and resistant strains being absorbed by crops and passed to humans through plant meals like vegetables [55]. Various studies [56–58] reported a noticeable amount of antimicrobial residues in animal and poultry manures used in agriculture production in various parts of the world. The findings point to the need for immediate strategies and activities to raise awareness and educate farmers about the problem, which might lead to their complete engagement in mitigation initiatives and hence protection against the health impacts of antimicrobial residues in foods [11].

The high frequency of using well water for vegetable irrigation could be due to its accessibility and cost-effectiveness [59]. However, most farmers’ inability to keep domestic animals from accessing water sources suggests that animal excrement containing antimicrobial residues could contaminate water sources as previously reported [60]. When contaminated water is used in vegetable cultivation regularly, the possibility of antimicrobial residues being absorbed by plant foods increases [10], putting the population’s health at risk upon consumption. However, more research is needed to determine the magnitude and scope of public exposure to antimicrobial chemicals in irrigation water from various sources in Dodoma city.

## Conclusion

In a view of the findings, about 42% of the consumers of eggs and Chinese cabbage in Dodoma city were generally not aware of the possibility of the antimicrobial residues in these foods. Specifically, the majority were unaware of the likelihood of antimicrobial residues in vegetables and their prevention methods. Education and location of the participants had significant effects on awareness where graduates and urban consumers were 7.7 and 1.6 times more aware than their uneducated and peri-urban counterparts. As for production practices, most farmers kept more layers than broilers and tetracyclines were the most used antimicrobial agents mainly for treatment and prophylaxis. Furthermore, the animal wastes were used as manure to amend the soil used for cultivation with the possibility of being absorbed by vegetables at higher concentrations. Therefore, reinforcing laws and regulations on antimicrobial use in food production and food safety measures alongside creating awareness and educating consumers in the city on antimicrobial residues in foods should be advocated. This will in turn serve as one of the mitigation strategies for consumer protection against unsafe foods and the associated health-related effects.

## Supporting information

S1 Data Dataset on animal production practices. (SAV)

S2 Data Dataset on vegetable production practices. (SAV)

S3 Data S3 Dataset on consumer awareness. (SAV)

S1 File Consent form. (PDF)

S2 File Questionnaire and checklists. (PDF)
