# Supplementary material for: Consumer awareness and production practices of farmers on antimicrobial residues in chicken eggs and Chinese cabbage in Dodoma, Central Tanzania
Source: PLoS One. 2022 Aug 18;17(8):e0272763. doi: 10.1371/journal.pone.0272763 (PMC9387843; doi:10.1371/journal.pone.0272763)
Supplement: S1 File — (PDF) [file pone.0272763.s004.pdf]

THE UNIVERSITY OF DODOMA

FOODBORNE ANTIMICROBIAL RESISTANCE RESEARCH

A Consent Form

Dear sir/madam

We the researchers from the Department of Public health and community Nursing of the University of Dodoma (UDOM) are conducting research titled “***Tackling Foodborne Antimicrobial Resistance: Assessment of Consumers’ Awareness and Levels of Antibiotic Residues in Raw and Cooked Chicken Eggs, Carrots and Chinese cabbage from Different Purchasing Sources in Dodoma City-Tanzania”.*** The main objective of the study is to establish consumer awareness, levels of antimicrobial residues in the mentioned foods and the effect of processing heat on the residues to strategize on mitigation plans

This research has taken into consideration all required procedures and has obtained permits from the office of the Vice Chancellor of UDOM and Dodoma Regional Commissioner and City Executive Officer. Therefore, as a resident of this city and a user of these foods, I kindly request you to participate in the study by filling this questionnaire. Should you accept our request and participate in this study then you will be interviewed by a trained research assistant and the information provided will strictly remain confidential between a researcher and respondent. Kindly be informed that no direct payment will be the effect upon your participation and the researcher reserve your free right to withdraw from the study anytime you wish or deem necessary to do so. Please, do not hesitate to ask just in case something is not clear or seems to be doubtful. Many thanks

Dr. Richard John Mongi

A lead Researcher

Statement of Consent

I have read and understood the purpose of the study and I voluntarily agree to participate in it.

Participant’s Signature…………………………………………. Date…………………………..

Researcher’s Signature…………………………………………. Date…………………………..
