# Supplementary material for: Consumer awareness and production practices of farmers on antimicrobial residues in chicken eggs and Chinese cabbage in Dodoma, Central Tanzania
Source: PLoS One. 2022 Aug 18;17(8):e0272763. doi: 10.1371/journal.pone.0272763 (PMC9387843; doi:10.1371/journal.pone.0272763)
Supplement: S2 File — (PDF) [file pone.0272763.s005.pdf]

THE UNIVERSITY OF DODOMA

FOODBORNE ANTIMICROBIAL RESISTANCE RESEARCH

A QUESTIONNAIRE

Introduction and Consent

Dear sir/madam

We the researchers from the Department of Public health and community Nursing of the University of Dodoma (UDOM) are conducting research titled "***Tackling Foodborne Antimicrobial Resistance: Assessment of Consumers' Awareness and Levels of Antibiotic Residues in Raw and Cooked Chicken Eggs, Carrots and Chinese cabbage from Different Purchasing Sources in Dodoma City-Tanzania".*** The main objective of the study is to establish consumer awareness, levels of antimicrobial residues in the mentioned foods and the effect of processing heat on the residues to strategize on mitigation plans

This research has taken into consideration all required procedures and has obtained permits from the office of the Vice Chancellor of UDOM and Dodoma Regional Commissioner and City Executive Officer. Therefore, as a resident of this city and a user of these foods, I kindly request you to participate in the study by filling this questionnaire. Should you accept our request and participate in this study then you will be interviewed by a trained research assistant and the information provided will strictly remain confidential between a researcher and respondent. Kindly be informed that no direct payment will be the effect upon your participation and the researcher reserve your free right to withdraw from the study anytime you wish or deem necessary to do so. Please, do not hesitate to ask just in case something is not clear or seems to be doubtful. Many thanks

Dr. Richard John Mongi

A lead Researcher

Statement of Consent

I have read and understood the purpose of the study and I voluntarily agree to participate in it.

Participant's Signature……………………………………… Date…………………………..

Researcher's Signature……………………………………… Date…………………………..

Appendix 1. Consumer Awareness questionnaire

Section 1: Demographic Information

1). Sex

[i]Male……. ii Female….

2). How old are you?

[i] 15- 20 [ii] 21-30 [iii] 31-40 [iv] 40 and above

3). What is the highest level of education you have attended?

[i] None [ii] Primary [iii] Secondary [iv] High school [v] College/University [vi] vocational

4). What is your religion?

[i] Christian [ii] Muslim [iii] Traditional [iv] Other specify

5) What is your current occupation?

[i] Peasant [ii] Business [iii] student [iv] Others…. (specify)

6). What is your marital status?

[i] Single [ii] married [iii] Widowed [iv] Divorced

Section 2 Consumption Practices

7). Where do you buy raw eggs, carrots and Chines cabbage for consumption?

[i] Local market [ii] Super market [iii] Farm [iv] shop

8. a] How often do you consume raw/cooked eggs, carrots and Chinese cabbages?

State (number of times/day/month/year) [i] eggs……………[ii] Carrot ………………………

b] Where often do you consume cooked eggs, carrots and Chinese cabbages?

[i]at home [ii] Hotels/Restaurants [iii] Street vendors (Bus stand/mama lishe/vibanda chips)

9. How do you prepare eggs and carrots for consumption?

[i] Raw [ii] Fried/roasted [iii] Boiled

Section 3: Consumers' Awareness of Drugs Residue in Eggs, Carrots and Chinese Cabbages

10. Do you know about drugs residue in eggs & carrots?

[i] Yes [ii] No

11. Have you ever heard about drugs residue in food of animal and plants origin?

[i] Yes [ii] No

12. Do you think drugs residues can be harmful to a human?

[i] Yes [ii] No

13. Do you know that animals are treated with antimicrobial drugs when are sick?

[i] Yes [ii] No

14. Do you know that animal waste/manure containing antimicrobial drugs is used for cultivating fruits and vegetables?

[i] Yes [ii] No

14. a) Do you know any common antimicrobial agent which can cause drugs residue in food of plants and animals origins like eggs, carrots and Chinese cabbage?

[i] Yes… ii] No…….

b) If the above 14. (a) is yes, please can you mention any antimicrobial drug (agent) that you know?

[i]…………………….. ii] ……………………… iii]………………………

15. a) Do you how to prevent drug residue in food like eggs and carrots?

[i] Yes [ii] No

b). If the above 15. (a) is yes, can you state methods used to prevent drugs residue in food like eggs carrots and Chinese cabbage? [i]………………. ii]………………………. iii]………

16. Those are all of the questions that I had, please do you have any comments that we haven't discussed above?……………………………………………………………

Many Thanks For Your Cooperation

Appendix 2. Checklist for Famers' Production Practices

1. Eggs Production

1.1 Farming Characteristics

1	What type of breed of Poultry do you keep?	a) Broilers
		b) Layers
		c) Others
2	How many birds do you have?	....
3	What type of ranging style have you adopted?	a) Intensive system
		b) Extensive system
3	How do you dispose off poultry Waste?	a) Use as manure
		b) Around the poultry farm
		c) Inside the stream
		d) Others

1.2. Pattern of antibiotics use in farms

SN	Question	Response
1	Do you use antimicrobial agents on Farm	a) Yes
		b) No
2	What types of antimicrobial agents do you use	a) Penicillin
		b) Amoxicillin
		c) Ampicillin
		d) Cloxacillin
		e) Augmentin
		f) Tetracycline
		g) Oxytetracycline
		h) Doxycycline
		i) Streptomycin
		j) Gentamicin
		k) Erythromycin
		l) Neomycin
		m) Chloramphenicol
		n) Cotrimoxazole
		o) Metronidazole
		p) Nalidixic Acid
		q) Other (specify)
3	Where do you get Information about antimicrobial agents you have been using?	a) Veterinary doctor
		b) By self
		c) Animal health worker
		d) Through the seller
		e) Through a friend
		f) Other (specify)
4	What are the reasons for antimicrobial usage?	a) To prevent and treat disease
		b) To prevent diseases

		c) To treat diseases
		d) Promote the growth of birds
		e) To prevent, treat and promote growth
		f) Other specify
5	Method of administration to Poultry	a) Through water
		b) Through water and injection
		c) Through water and food
		d) Through injection
		e) Through water, food and injection
6	Compliance with a withdrawal period	a) Yes
		b) No
7	Frequency of Use of antimicrobial agent	a) Daily-once a week
		b) Once in two weeks- once a month
		c) When they are sick
		d) Others (specify)

2. Vegetables production

SN	Water usage, soil and manure usage	Responses
1	What is the source of irrigation water?	a) Pond
		b) Stream
		c) Well
		d) Municipal
		e) Other) please specify
2	How are crops irrigated?	a) Flood
		b) Drip
		c) Sprinkler
		d) Other) please specify
3	Water quality is known to be adequate for the crop irrigation method and the crop being irrigated.	a) Yes
		b) No
4	Water quality is known to be adequate for chemical application or fertigation methods.	a) Yes
		b) No
5	If necessary, steps are taken to protect irrigation water from potential direct and non-point source contamination.	a) Yes
		b) No
6	The farm sewage treatment system/septic system is functioning properly and there is no evidence of leaking or runoff.	a) Yes
		b) No
7	There is no municipal/commercial sewage treatment facility or waste material landfill adjacent to the farm.	a) Yes
		b) No
8	Crop production areas are not located near or adjacent to dairy, livestock, or fowl production facilities.	a) Yes
		b) No
9	Manure lagoons located near or adjacent to crop	a) Yes

	production areas are maintained to prevent leaking or overflowing, or measures have been taken to stop runoff from contaminating the crop production areas.	b) No
10	Manure stored near or adjacent to crop production areas is contained to prevent contamination of crops.	a) Yes<br>b) No
11	Measures are taken to restrict access of livestock to the source or delivery system of crop irrigation water.	a) Yes<br>b) No
12	Measures are taken to reduce the opportunity for wild and/or domestic animals to enter crop production areas.	a) Yes<br>b) No
13	Crop production areas are monitored for the presence of signs of wild or domestic animals entering the land.	a) Yes<br>b) No
14	Please choose one of the following options as it relates to the farm operation	a) Raw manure or a combination of raw and composed manure is used as a soil amendment.<br>b) Only composted manure/treated municipal biosolids are used as a soil amendment.<br>c) No manure or municipal biosolids of any kind are used as a soil amendment.
15	Previous land use history indicates that there is a minimum risk of produce contamination with antimicrobials	a) Yes<br>b) No
16	Previous land use history indicates a possibility of contamination and preventative measures have been taken to mitigate the known risks and soils have been tested for contaminants and the land use is commensurate with test results.	a) Yes<br>b) No
17	Crop production areas that have been subjected to flooding are tested for potential antimicrobial microbial hazards.	a) Yes<br>b) No

Adapted from Kline et al. (2012)

N.B. No data was collected in carrot as the vegetable is not cultivated in Dodoma city
